# Analysis of lactating cows on commercial Austrian dairy farms: the influence of genotype and body weight on efficiency parameters

**DOI:** 10.5194/aab-62-491-2019

**Published:** 2019-07-29

**Authors:** Maria Ledinek, Leonhard Gruber, Franz Steininger, Birgit Fuerst-Waltl, Karl Zottl, Martin Royer, Kurt Krimberger, Martin Mayerhofer, Christa Egger-Danner

**Affiliations:** 1 Department of Sustainable Agricultural Systems, BOKU – University of Natural Resources and Life Sciences Vienna, Vienna, 1180, Austria; 2 Agricultural Research and Education Centre Raumberg-Gumpenstein, Irdning-Donnersbachtal, 8952, Austria; 3 ZuchtData EDV-Dienstleistungen GmbH, Vienna, 1200, Austria; 4 LKV Niederösterreich, Zwettl, 3910, Austria

## Abstract

The aim of this study was twofold: first, to evaluate the influence of body weight on the efficiency of dairy cows, and second, to analyze the current state of dairy cattle populations as part of the Austrian Cattle Breeding Association's Efficient Cow project.

Data of Fleckvieh (FV, dual-purpose Simmental), Fleckvieh×Red Holstein (FV×RH), Holstein (HF) and Brown Swiss (BS) dairy cows (161 farms, 6098 cows) were collected at each performance recording during the year 2014.

In addition to routinely recorded data (e.g., milk yield, fertility), body weight, body measurements, body condition score (BCS) and individual feed information were also collected. The following efficiency traits were considered: body weight efficiency as the ratio of energy-corrected milk (ECM) to metabolic body weight, feed efficiency (kilogram ECM per kilogram dry-matter intake) and energy efficiency expressed as the ratio of energy in milk to energy intake.

The relationship of milk yield to body weight was shown to be nonlinear. Milk yield decreased in cows above the 750 kg body weight class for HF, BS and FV×RH with 68 % RH genes, but less dramatically and later for FV at 800 kg. This resulted in an optimum body weight for feed and energy efficiency. BS and HF had the highest efficiency in a narrower and lighter body weight range (550–700 kg) due to a stronger curvature of the parabolic curve. Contrary to this, the efficiency of FV did not change as much as it did in the dairy breeds with increasing body weight, meaning that FV had a similar feed and energy efficiency in a range of 500–750 kg. The breed differences disappeared when body weight ranged between 750 and 800 kg.

The average body weight of the breeds studied (FV 722 kg, BS 649 and HF 662 kg) was in the optimum range. FV was located at the upper end of the decreasing segment.

In conclusion, an optimum body weight range for efficiency does exist, due to the nonlinear relationship of milk yield and body weight. Specialized dairy breeds seem to respond more intensively to body weight range than dual-purpose breeds, due to the stronger curvature. Cows with medium weights within a population are the most efficient. Heavy cows (>750 kg) produce even less milk. A further increase in dairy cows' body weights should therefore be avoided.

## 1 Introduction

Over the last decades, milk performance has increased dramatically and resulted in a decline in fertility, vitality and longevity (Knaus, 2009). This development has reduced cows' cost effectiveness. In the USA, Bavaria (Germany) and Austria (Knaus, 2009), the number of completed lactations has dropped under the calculated critical threshold of four parities (Essl, 1982). In Austria, there have been efforts to stop this trend, including introducing a breeding value for longevity in 1995 and a joint genetic evaluation in Austria and Germany in 2002 (Fuerst and Egger-Danner, 2002). Cows' body size is also increasing. In the USA, Holstein cows were selected directly for body size to some extent, on the assumption that larger cows are able to produce more milk (Hansen, 2000). In Bavaria (Germany), increasing body size in Fleckvieh (dual-purpose Simmental) and Brown Swiss (BS) has been negatively connected to longevity (Krogmeier, 2009). In 1966, a long-term experiment with Holstein (HF) cows at the Northwest Experiment Station, University of Minnesota, concerning cow size was initiated and resulted in several studies (e.g., Mahoney et al., 1986; Hansen et al., 1999; Becker et al., 2012). The selected line became larger and heavier but had higher health costs. Studies, for example Brown et al. (1977), have shown that the highest milk yield was reached in the medium body weight range; large and heavy cows were not found to be at an advantage, neither in health and fertility traits, nor in milk production. The relevant genetic relationship between milk yield and body weight is difficult to quantify and varies due to the distorting effects of body tissue mobilization and a lack of sufficient data, but it is assumed to be positive (Veerkamp, 1998). However, heavier cows have to produce more milk to be as efficient as lighter cows to dilute the negative effect of their high body weight and therefore increased maintenance requirements (Hansen et al., 1999; Steinwidder, 2009). In countries like Ireland and New Zealand, where dairy cows are bred for the efficient use of pasture, animals are lighter and have a higher body condition but produce approximately only half of the milk yield (Knaus, 2016).

The Federation of Austrian Cattle Breeders (ZAR) initiated the project Efficient Cow in 2012 to develop efficiency traits for Austrian cattle breeding. Within this framework, the aims of this study were (1) to examine the influence of body weight and genotype on different efficiency parameters for milk production, (2) to clarify if an optimum body weight for highest efficiency exists and to describe the current state of the examined dairy cattle population, and (3) to give recommendations concerning body weight in cattle breeding.

## 2 Materials and methods

### 2.1 Data recording and calculation

During a 1-year recording period in 2014, data from 3628 Fleckvieh (FV) and FV×RH (Red Holstein), 1034 HF, and 1436 BS cows kept on 161 dairy farms in Austria were collected. Cows were mostly housed in free-stall barns and milked twice a day in a milking parlor. The Austrian milk recording organizations collected new traits like body weight with a mobile scale, body condition score (BCS), body measurements, and information about diet and diet quality for each routine performance recording day. Data were stored in the Austrian central cattle database. Forage was sampled either before feeding or at the start of the project. Samples were taken separately according to conservation method, harvest number (first cutting separately) and botanic origin. On average, the farms had 9.8 milk recordings per year, ranging between 9 and 11 times. The number of reports per cow ranged from 1 to 12 with a mode of 8 and a mean of 6.2 reports. Farms were located between 300 and 1460 m above sea level in flat, hilly and mountainous areas (Ledinek et al., 2019a, b). Herd sizes varied between 3.2 and 97.9 cows, reflecting the wide range of herd size in Austria. The production level and average herd size of the project farms (32.7 cows) were above average compared to other Austrian farms with 16.5 cows (ZAR, 2016).

The handling of forage analyses (VDLUFA, 1976), nutrient content of concentrate (DLG, 1997) and calculation of energy content of forage (GfE, 2001) have been described in detail in a previous article by Ledinek et al. (2019a). The laboratory for feed analyses of the Chamber of Agriculture in Lower Austria analyzed the forage samples using Weende analysis and the method described by Van Soest et al. (1991). Dry-matter intake (DMI) was estimated because comprehensively measuring feed intake on-farm was not feasible (Gruber et al., 2004; Ledinek et al., 2016). This situation provided the opportunity to develop novel strategies for recording diet composition information on-farm. Feeding system and diet composition were also considered in the feed intake prediction model. The prediction model selected for this study was found to be the most valid and accurate model in a comparison of four up-to-date models (Jensen et al., 2015). A detailed description of recording diet information, feed intake estimation and the results of diet composition can be found in Ledinek et al. (2016) and Ledinek et al. (2019a). Energy-corrected milk (ECM) was calculated according to the recommendations of GfE (2001). Body condition was evaluated using the five-point system by Edmonson et al. (1989).

As recommended by Berry and Pryce (2014), efficiency parameters were calculated as the ratio between output and input and named after the input parameter in the current study. The estimation of feed intake resulted in the exclusion of residual feed intake. Body weight efficiency was calculated as kilogram ECM per kilogram metabolic body weight (BW${}^{0.75}$), feed efficiency as kilogram ECM per kilogram DMI and energy efficiency as energy in milk (LE) per energy intake, both expressed in megajoule of net energy for lactation (NEL). Therefore, energy efficiency takes both diet quality and concentrate proportion into account. This study focused solely on efficiency in dairy production. Considering additional aspects relevant to a holistic (economic) comparison of different genotypes like fattening potential, health or fertility would have exceeded the scope of this study.

### 2.2 Statistical analysis

The data set during lactation included 37 967 records (milk performance recordings), 161 farms and 6098 cows.

Combined genotype–body-weight classes were established to cover differing body weight ranges within the genotypes. Body weight classes were set at 50 kg intervals from 450 to 1000 kg. Cows with a body weight between ≥425 and <475 kg were put in the 450 kg class, cows weighing between ≥475 and <525 kg were in the 500 kg class, etc. The name of each class therefore reflects the average body weight of the animals in that class.

Fleckvieh (100 % FV ancestry, 1575 cows), Fleckvieh with an average of 25 % RH genes (FV×RH25, 404 cows) and Fleckvieh with an average of 68 % RH genes (FV×RH5075, 345 cows) were in body weight classes ranging from 500 to 950 kg. Fleckvieh with an average of 6.25 % RH genes (FV×RH6.25, 963 cows) were in body weight classes of 500–1000 kg, and Fleckvieh with an average of 12.5 % RH genes (FV×RH12.5, 341 cows) were in the body weight classes from 550 to 950 kg. The body weight classes of 450–900 kg were included for the lighter BS and HF cows (both 100 % ancestry of the respective breed, 1436 and 1034 cows). Lower and higher weight classes were discarded due to insufficient numbers of animals in the respective classes.

The stage of lactation consisted of twelve 28 d stages from 1 to 336 d in milk (DIM).

The following final model for dependent traits (e.g., DMI, BCS, efficiency traits) was used:

1 $$\begin{array}{rl} Y_{ijklm} & =\mu +G\_{\mathrm{BW}}_{i}+P_{j}+{\mathrm{SL}}_{k}+F_{l}+b_{\mathrm{NELFor}}\\ & \times \;\mathrm{NELFor}+b_{\mathrm{Conc}}\times \mathrm{Conc}+{\mathrm{Cow}}_{m}\left(F_{l}\right)+\varepsilon_{ijklm}, \end{array}$$

where $Y_{ijklm}$ is trait, μ is the intercept, $G\_{\mathrm{BW}}_{i}$ is the fixed effect of genotype–body-weight class (1–70), $P_{j}$ is the fixed effect of parity (1, 2, 3+4, ≥5), ${\mathrm{SL}}_{k}$ is the fixed effect of lactation stage (1–12), $F_{l}$ is the fixed effect of farm (1–161), $b_{\mathrm{NELFor}}$ is the linear regression on energy content of forage (NELFor), $b_{\mathrm{Conc}}$ is the linear regression on concentrate proportion (Conc), Cow${}_{m}\left(F_{l}\right)$ is the random effect of cow nested within farm and $\varepsilon_{ijklm}$ is the residual.

Traits were analyzed using PROC MIXED of SAS 9.4 (SAS, 2015), the restricted maximum likelihood (REML) method, the Kenward–Roger method and the covariance structure Variance Components (VC) causing the smallest Akaike information criterion.

## 3 Results

**Figure 1:**
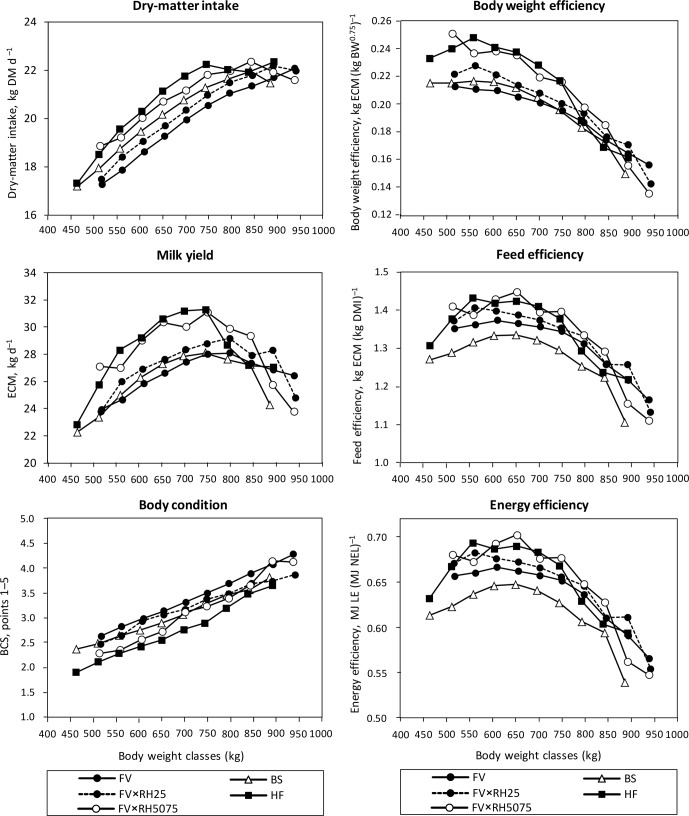
Effect of body weight on feed intake, milk production, BCS, body weight efficiency, feed efficiency and energy efficiency (LE: energy in milk) of Brown Swiss (BS), Fleckvieh (FV), the selected FV groups with increasing Red Holstein (RH) genes FV×RH25 and FV×RH5075 as well as Holstein Friesian (HF).

Table 1 contains BCS, milk production, estimated DMI and energy intake, while DMI per kilogram body weight and efficiency parameters can be found in Table 2. The root mean square errors are shown separately (Table 3). Apart from energy content of forage on BCS (P=0.145), all effects included in the statistical model influenced all dependent traits significantly (P<0.001). The efficiency parameters, DMI, ECM and BCS of selected genotypes are shown in Fig. 1.

**Table 1:** Effect of genotype×body weight on BCS, energy-corrected milk, feed and energy intake (least squares means).

Trait${}^{\text{a}}$	Genotype${}^{\text{b}}$	Body weight classes (450–1000 kg)											
		450	500	550	600	650	700	750	800	850	900	950	1000
Data set (N=37967)		81	680	2151	5039	7731	8170	6668	4301	2061	832	232	21
Body condition, points 1–5													
	FV		2.63	2.82	2.99	3.13	3.31	3.49	3.68	3.89	4.07	4.28	
	FV×RH6.25		2.54	2.65	2.91	3.08	3.28	3.46	3.63	3.81	4.07	4.24	4.49
	FV×RH12.5			2.89	2.98	3.11	3.22	3.39	3.59	3.77	3.92	4.39	
	FV×RH25		2.47	2.64	2.93	3.06	3.16	3.37	3.48	3.69	3.73	3.86	
	FV×RH5075		2.28	2.35	2.56	2.72	3.12	3.23	3.39	3.65	4.14	4.12	
	HF	1.90	2.11	2.28	2.42	2.55	2.76	2.89	3.19	3.47	3.64		
	BS	2.37	2.48	2.64	2.75	2.89	3.05	3.28	3.46	3.56	3.80		
ECM, kg d${}^{-1}$													
	FV		23.9	24.6	25.8	26.6	27.4	28.0	28.1	27.4	26.8	26.4	
	FV×RH6.25		23.7	24.9	25.8	26.8	27.4	28.4	28.2	27.4	26.1	24.2	24.0
	FV×RH12.5			25.7	25.1	26.8	28.5	28.5	28.8	27.8	25.6	24.8	
	FV×RH25		23.8	26.0	26.9	27.6	28.3	28.8	29.2	27.9	28.3	24.8	
	FV×RH5075		27.1	27.0	29.0	30.4	30.0	31.1	29.9	29.3	25.7	23.8	
	HF	22.8	25.7	28.3	29.2	30.6	31.2	31.3	28.7	27.2	27.1		
	BS	22.2	23.4	25.0	26.3	27.3	27.9	28.1	27.6	27.2	24.2		
Dry-matter intake, kg DM d${}^{-1}$													
	FV		17.28	17.87	18.62	19.27	19.96	20.55	21.05	21.35	21.70	22.07	
	FV×RH6.25		17.47	17.99	18.68	19.36	19.93	20.64	21.09	21.39	21.75	21.50	22.09
	FV×RH12.5			18.29	18.59	19.45	20.30	20.76	21.28	21.46	21.70	21.78	
	FV×RH25		17.48	18.41	19.07	19.69	20.35	20.97	21.50	21.79	22.17	21.98	
	FV×RH5075		18.86	19.21	20.04	20.71	21.17	21.82	21.97	22.36	21.93	21.59	
	HF	17.31	18.52	19.57	20.29	21.14	21.76	22.24	22.04	21.93	22.35		
	BS	17.20	17.93	18.76	19.47	20.16	20.75	21.28	21.65	21.97	21.47		
Energy intake, MJ NEL d${}^{-1}$													
	FV		114.7	118.7	123.5	127.5	132.1	136.0	139.3	141.1	143.3	145.6	
	FV×RH6.25		116.3	119.7	123.8	128.2	132.0	136.5	139.6	141.5	143.5	142.0	145.6
	FV×RH12.5			121.7	123.0	128.8	134.4	137.4	140.8	141.8	144.0	143.3	
	FV×RH25		115.8	121.8	126.3	130.4	134.7	138.8	142.3	144.3	146.7	145.2	
	FV×RH5075		125.1	127.0	132.7	137.1	140.2	144.5	145.3	147.8	145.0	142.6	
	HF	114.5	122.6	129.4	134.3	140.1	144.2	147.4	145.9	145.1	148.1		
	BS	115.1	119.2	124.5	129.0	133.5	137.3	140.9	143.4	145.4	141.7		

${}^{\text{a}}$ ECM: energy-corrected milk (GfE, 2001); DM: dry matter; NEL: net energy for lactation.${}^{\text{b}}$ FV: Fleckvieh; RH: Red Holstein; 6.25–25: average proportion of Red Holstein; FV×RH5075: Fleckvieh with an average proportion of 68 % Red Holstein; HF: Holstein Friesian; BS: Brown Swiss.

**Table 2:** Effect of genotype×body weight on dry-matter intake per kilogram body weight and efficiency parameters (least squares means).

Trait${}^{\text{a}}$	Genotype${}^{\text{b}}$	Body weight classes (450–1000 kg)											
		450	500	550	600	650	700	750	800	850	900	950	1000
Data set (N=37967)		81	680	2151	5039	7731	8170	6668	4301	2061	832	232	21
Dry-matter intake, g (kg BW${}^{0.75}{)}^{-1}$													
	FV		156.0	153.3	151.3	148.7	146.2	143.3	140.1	136.0	132.9	130.4	
	FV×RH6.25		157.1	154.5	152.4	149.6	146.2	144.0	140.5	136.7	132.8	127.0	126.2
	FV×RH12.5			158.2	151.8	150.3	148.7	144.6	141.6	136.6	132.1	128.0	
	FV×RH25		159.9	160.2	156.3	152.2	149.3	146.3	142.9	138.1	134.5	127.4	
	FV×RH5075		174.2	167.4	164.1	160.4	155.2	152.1	145.9	141.9	133.6	124.9	
	HF	173.6	171.6	170.7	166.9	164.2	159.9	155.2	146.1	137.9	134.2		
	BS	167.0	164.3	162.3	159.5	156.4	152.8	149.0	144.2	139.7	131.9		
Body weight eff., kg ECM (kg BW${}^{0.75}{)}^{-1}$													
	FV		0.213	0.211	0.210	0.205	0.201	0.195	0.187	0.174	0.165	0.156	
	FV×RH6.25		0.210	0.213	0.211	0.208	0.201	0.198	0.188	0.175	0.159	0.145	0.139
	FV×RH12.5			0.222	0.206	0.208	0.208	0.199	0.192	0.177	0.155	0.146	
	FV×RH25		0.222	0.228	0.221	0.214	0.208	0.201	0.194	0.176	0.171	0.143	
	FV×RH5075		0.251	0.237	0.238	0.235	0.220	0.216	0.198	0.185	0.156	0.136	
	HF	0.233	0.240	0.248	0.241	0.238	0.228	0.217	0.188	0.169	0.162		
	BS	0.215	0.215	0.217	0.216	0.212	0.205	0.196	0.183	0.172	0.150		
Feed efficiency, kg ECM (kg DMI)${}^{-1}$													
	FV		1.351	1.361	1.373	1.365	1.356	1.344	1.312	1.257	1.216	1.163	
	FV×RH6.25		1.321	1.360	1.366	1.368	1.355	1.359	1.316	1.257	1.179	1.102	1.064
	FV×RH12.5			1.389	1.337	1.364	1.381	1.352	1.330	1.272	1.135	1.109	
	FV×RH25		1.371	1.406	1.398	1.387	1.374	1.352	1.332	1.258	1.257	1.131	
	FV×RH5075		1.409	1.387	1.429	1.448	1.395	1.396	1.334	1.291	1.154	1.109	
	HF	1.306	1.377	1.431	1.418	1.424	1.410	1.377	1.292	1.237	1.218		
	BS	1.270	1.287	1.315	1.333	1.334	1.321	1.294	1.252	1.222	1.104		
Energy efficiency, MJ LE (MJ NEL)${}^{-1}$													
	FV		0.657	0.660	0.666	0.662	0.658	0.652	0.637	0.610	0.591	0.566	
	FV×RH6.25		0.639	0.658	0.662	0.663	0.657	0.660	0.639	0.610	0.573	0.533	0.514
	FV×RH12.5			0.669	0.648	0.662	0.669	0.656	0.645	0.617	0.549	0.537	
	FV×RH25		0.671	0.683	0.676	0.672	0.666	0.656	0.646	0.610	0.611	0.554	
	FV×RH5075		0.680	0.673	0.692	0.702	0.676	0.677	0.648	0.628	0.562	0.547	
	HF	0.632	0.667	0.694	0.687	0.690	0.684	0.668	0.629	0.604	0.594		
	BS	0.614	0.623	0.637	0.646	0.647	0.641	0.627	0.606	0.594	0.539		

${}^{\text{a}}$ BW${}^{0.75}$: metabolic body weight; ECM: energy-corrected milk (GfE, 2001); DMI: dry-matter intake; LE: energy in milk, NEL: net energy for lactation.${}^{\text{b}}$ FV: Fleckvieh; RH: Red Holstein; 6.25–25: average proportion of Red Holstein; FV×RH5075: Fleckvieh with an average proportion of 68 % Red Holstein; HF: Holstein Friesian;BS: Brown Swiss.

**Table 3:** Root mean square error of efficiency and production traits.

Trait${}^{\text{a}}$	RMSE${}^{\text{b}}$
Body condition, points 1–5	0.4
ECM, kg d${}^{-1}$	5.5
Dry-matter intake, kg DM d${}^{-1}$	1.16
Energy intake, MJ NEL d${}^{-1}$	7.9
Dry-matter intake, g (kg BW${}^{0.75}{)}^{-1}$	8.3
Body weight eff., kg ECM (kg BW${}^{0.75}{)}^{-1}$	0.040
Feed efficiency, kg ECM (kg DMI)${}^{-1}$	0.208
Energy efficiency${}^{\text{b}}$, MJ LE (MJ NEL)${}^{-1}$	0.102

${}^{\text{a}}$ ECM: energy-corrected milk (GfE, 2001); DM: dry matter; NEL: net energy for lactation; BW${}^{0.75}$: metabolic body weight; DMI: dry-matter intake; LE: energy in milk.${}^{\text{b}}$ Root mean square error.

Average milk production, DMI and efficiency parameters increased for the most part continuously, together with rising RH genes from FV to HF as previously described in detail (Ledinek et al., 2019a, b). BS had a lower feed and energy efficiency than FV. For most traits, BS came in between the two genotypes FV and HF.

Feed and energy intake increased up to 750 kg body weight and then tended to stagnate or even to decline, especially in the genotypes with a high proportion of specialized dairy breeds (FV×RH5075, HF and BS). Furthermore, genotypes were similar in the very light and the very heavy body weight classes. This development was found to be even stronger when DMI was calculated relative to body weight. The most significant change and therefore the strongest curvature again occurred in the specialized dairy genotypes. A look at milk production (Fig. 1) could explain this pattern: the highest production was reached not in the heaviest body weight classes but in the medium ones. The FV groups with up to an average of 25 % RH genes reached the ECM peak at 800 kg, except for FV×RH6.25, the lighter FV×RH5075, HF and BS, which peaked earlier at 750 kg. After the peak, the decline in milk yield together with rising body weight continued and differences between dual-purpose types and dairy types vanished.

This pattern was also observed for efficiency parameters, although body weight efficiency differed from feed and energy efficiency. HF produced the most ECM per kilogram body weight in the 500–650 kg range; BS does so in the lightest classes at 450–650 kg. In contrast, the efficiency of FV and FV×RH6.25 declined slightly from the start but remained on a similar level until reaching the 650 kg body weight class. The FV groups with up to an average of 12.5 % RH genes showed the lowest loss of body weight efficiency with increasing weight.

Although breed differences in feed and energy efficiency vanished again at a weight of 800 kg, peak efficiency shifted to cows with medium weight. The optimum range of BS and HF was 550–700 kg, peaking between 550 and 650 kg. Contrary to this, the efficiency of FV remained steady from 500 to 750 kg, with the highest efficiency at 600 kg. Efficiency declined increasingly, and was observable starting from the body weight classes of 750–800 kg.

Body condition (Fig. 1) rose in a nearly linear fashion with increasing body weight. In the optimum range, FV had a BCS of 2.63–3.49 points, HF had one of 2.28–2.76 points and BS one of 2.64–3.05 points.

## 4 Discussion

The nonlinear relationship between milk yield and body weight and the stronger curvature of the specialized dairy groups (FV×RH5075, BS and HF) give rise to the question of how these traits are connected to each other.

In the current study, body weight and milk yield were phenotypically correlated to a low degree of 0.12. Due to the nonlinear relationship, it would be a mistake to assume a failing connection between the two traits. Enevoldsen and Kristensen (1997) found nearly nonexistent negative and positive correlations within Red Danish×Jerseys, Jerseys and Danish Friesians. Veerkamp et al. (2000) found slightly higher positive phenotypic relationships in the first 15 weeks of lactation.

Published results on genetic relationships vary as well. In earlier studies (Mason et al., 1957; Hooven et al., 1968), positive relationships were reported, while Veerkamp (1998) revealed a range between -0.41 and 0.45 in his review. Veerkamp (1998) attributed this large variation to differences in data recording times and insufficient measurements in the included studies. Furthermore, the strong connection of body weight to BCS and therefore to mobilization dilutes the actual effect. After the genetic adjustment for BCS, the correlation between body weight and milk yield was reported to be medium positive (Veerkamp, 1998). This would confirm the findings of another review (Hansen, 2000) that it is not only milk production but also sharpness and body size of US Holstein cows that has increased over recent decades. In a long-term experiment concerning body size, large cows suffered more from claw and leg diseases due to their higher body weight (Hansen et al., 1999). Furthermore, a previous study within this project showed that larger cows required more health care (Mahoney et al., 1986). In Bavaria (Germany), BS and FV cows have increased in body size over the last decades (Krogmeier, 2009). Krogmeier (2009) also found a negative relationship between body size and longevity. Comparisons of body weight data in more recent Austrian studies (Gruber and Stegfellner, 2015; Ledinek et al., 2019a, b) with an older study (Haiger et al., 1987) seem to confirm an increasing trend in body weight in BS and HF. However, the body weight trend could also be affected by overlaying effects. For FV, the increasing selection for dairy traits may have resulted in cows with less muscle mass and BCS but perhaps with larger frames; on the other hand, crossbreeding with lighter Red Holstein was common.

In the current study, the phenotypic correlations between body weight and efficiency were -0.18 for body weight efficiency, -0.11 for feed efficiency and -0.13 for energy efficiency. Prendiville et al. (2009) reported a significantly stronger relationship with -0.46 to -0.50 for their efficiency parameters, which went along with a stronger correlation between body weight and milk yield in a similar range. Dickinson et al. (1969) found a relationship between body weight and energy efficiency of -0.27, and a relationship between energy efficiency and chest depth, heart girth and body length of between -0.21 and -0.38. Their observation that energy efficiency decreases linearly with body weight, while changes in heart girth and body weight cause a quadratic effect, was particularly interesting. Vallimont et al. (2011) confirmed these phenotypic findings with even stronger negative genetic correlations of various efficiency parameters from -0.64 to -0.66 and concluded that larger and fatter cows were less efficient. Furthermore, heavier cows have to produce more milk to be as efficient as lighter cows to dilute the effect of their increasing maintenance requirements (Steinwidder, 2009). As feed intake increases at a rate of 0.22 kg per additional kilogram milk (Gruber et al., 2004), heavier cows need a higher quality diet or they have to mobilize body tissue to reach an “efficient” milk yield (Steinwidder, 2009). Figure 1 shows that heavier cows have a lower feed intake per kilogram BW${}^{0.75}$ than lighter and medium-weight animals.

The finding that having medium-weight cows in a population is optimal has also been confirmed by much older studies (Hooven et al., 1968; Miller and Hooven, 1969; Brown et al., 1977). Hansen et al. (1999) compared lighter and heavier HF lines and concluded that due to frequent problems in health and fertility of heavy cows, an optimum body weight range may exist. As Fig. 1 shows, dry-matter intake and milk yield behave differently with regard to their correlation with body weight. Dry-matter intake does increase with increasing body weight over its total range but to a decreasing extent. Contrary to this, milk yield declines in high body weight classes. As a consequence, efficiency parameters reach their maximum not at the lowest or highest body weights, but somewhere in between, more towards the lower end of the weight range. The somatotropic axis controls nutrient partitioning between milk production and body tissue, mainly through the hormones somatotropin and the insulin-like growth factor (Lucy, 2000; Lucy et al., 2009). A high potential for milk production reduces body condition within and among breeds (e.g., Buckley et al., 2000; Dillon et al., 2003; Ledinek et al., 2019b). Consequently, the precondition for a large and heavy body (high body weight and BCS) is the genetic potential for partitioning nutrients into growth and body tissue to a higher extent. This explains why cows in the heavier body weight classes with concurrent higher BCS (Fig. 1) produced less milk relative to their weight. If they produced relatively more ECM, they would be large-framed dairy types with low BCS. Veerkamp (1998) described a negative genetic association between milk yield and BCS. After the genetic adjustment for BCS, the moderately positive genetic association between milk yield and body weight was in line with the positive relationships between milk yield and body size measurements.

In the current study, the higher the gene proportion of specialized dairy breeds, the more the genotypes responded to the range of body weight. Corresponding results were found in a study by Gruber et al. (2017) based on data from German and Austrian research institutes. Somatotropin and the insulin-like growth factor control many aspects of lactation, growth and fertility in cattle (Lucy, 2000). The selection for milk production changed the nutrient partitioning mechanisms (Lucy et al., 2009) due to the high metabolic priority given to milk production (Bauman and Currie, 1980). This suggests that the relationship between feed intake, ECM and body weight is not the same in dual-purpose and dairy breeds due to differing nutrient partitioning as shown in the current study. Resources like nutrients and energy are primarily put into performance in high-yielding dairy cows (Huber, 2018). It is not only inadequate management (Huber, 2018) but also an inadequate nutrient intake in the first third of lactation (Bauman and Currie, 1980) that limit available resources and make nutrients scarce, especially for maintenance (body, BCS), fertility and health (Huber, 2018). In specialized dairy cows, a small shift in priority towards body weight and BCS along the body weight range probably has a stronger effect on nutrient partitioning towards milk yield. This explains the increasing dependency of efficiency on body weight with increasing specialization for milk production in the current study. During lactation, high-yielding cows have higher levels of growth hormones, nonesterified fatty acids (NEFAs) and β-hydroxybutyric acid and lower levels of insulin in the blood (Hart et al., 1978). This is accompanied by increased body tissue mobilization. Unlike in low-yielding cows, levels of growth hormones and NEFA are significantly reduced in the dry period, while glucose levels rise. As a result, changes in, e.g., milk yield, body weight, endocrine and energy state are more pronounced in high-yielding than in lower-yielding cows.

These indications led to the assumption that the increasing body weight classes within a breed may reflect the spectrum of potential in dairy traits, with a higher sensitivity of dairy types to body weight range. However, it must be emphasized that for animals with a low BCS, it was not possible to differentiate between light dairy types and cows that had previously mobilized large amounts of body tissue.

Nevertheless, the groups with a higher proportion of specialized dairy breeds only benefited from their superiority in milk production in the medium body weight range as compared to the dual-purpose types. The average body weight of the FV groups with an average of up to 25 % RH genes was between 722 and 729 kg, and that of HF and BS was 662 and 649 kg, respectively, in the overall analysis (Ledinek et al., 2019a, b). Therefore, the lighter specialized dairy breed groups were actually at the peak of their optimum nutrient efficiency, while the FV groups with an average of up to 12.5 % RH genes as dual-purpose types were located on the upper end of the decreasing segment. FV×RH25 was found to exceed its optimum range between 500 and 700 kg due to the stronger curvature. The lighter groups HF, BS and FV×RH5075 were near the upper end of their optimum range of body weight efficiency. The groups FV, FV×RH6.25 and FV×RH25 exceeded their optimum range. Furthermore, another study conducted as part of the current project (Köck et al., 2018) revealed a medium positive genetic correlation between lameness and body weight. Heavier cows may have more problems with lameness than lighter ones.

## 5 Conclusions

The relationship between milk yield and body weight was found to be nonlinear. Heavy and very light cows produced less milk than cows of medium body weight. The nonlinear relationship between milk production and body weight resulted in an optimal body weight for highest feed and energy efficiency in the medium body weight range of the population. The specialized dairy breeds seemed to respond more intensively to body weight range than dual-purpose breeds. Their superiority in feed and energy efficiency was only observable in the medium body weight range within populations. In Austria, HF and BS have currently reached their optimum of nutrient efficiency. FV is still within the optimum range of body weight but is reaching the top end. As optimum body weight efficiency is located towards the lighter body weight range, all genotypes are too heavy as to be at the peak of optimum.

Therefore, further increases in body weight of all breeds with regard to nutrient and body weight efficiency cannot be recommended. A broader definition of efficiency including additional aspects like health, fertility or fattening potential should be investigated in the future.

## Data Availability

The data sets analyzed during the current study are not publicly available as information contained therein could compromise the privacy of third parties.
